# Lipid Peroxidation Products in Human Health and Disease 2016

**DOI:** 10.1155/2017/2163285

**Published:** 2017-02-28

**Authors:** Kota V. Ramana, Sanjay Srivastava, Sharad S. Singhal

**Affiliations:** ^1^Department of Biochemistry and Molecular Biology, University of Texas Medical Branch, Galveston, TX 77555, USA; ^2^Environmental Cardiology, University of Louisville, Louisville, KY 40202, USA; ^3^Department of Molecular Medicine, Beckman Research Institute, City of Hope National Medical Center, Duarte, CA 91010, USA

Lipid peroxidation has been implicated in the etiology of several diseases. The process of lipid peroxidation can be initiated by a variety of oxidants, including H_2_O_2_, superoxide, and the highly reactive hydroxyl radicals during pathological conditions or exposure to xenobiotics and environmental pollutants. Lipid peroxidation can alter vital membrane protein structure and function, and if unchecked, it could lead to cellular dysfunction and widespread tissue damage. Despite multiple studies showing that uncontrolled and excessive production of lipid peroxidation products during oxidative stress are the main cause of various disease complications, the mechanisms by which lipid peroxidation products regulate oxidative, immune, and inflammatory responses remain unclear. Therefore, understanding the role of various lipid peroxidation products in mediation of oxidative and inflammatory signaling is potentially important in developing better therapeutic strategies. In the series of special issues, we are continuously highlighting the significant role of lipid peroxidation products in human health and disease.

The 3 review articles published in this issue discussed how oxidative stress and lipid peroxidation products are involved in various pathological conditions. An excellent and informative review article by S. Q. Rodríguez-Lara et al. described the relationship between oxidative stress and ischemia/reperfusion (I/R) lesions. Specifically, they discussed how current pharmaceutical and mechanical interventions for I/R, although promising, cannot be used in all patients. Further, the significance of reactive oxygen species (ROS) and reactive nitrogen species (RNS) in the I/R has been exclusively discussed and suggested possible antioxidative stress therapeutic regimens in the treatment of I/R lesions. Another review article by J. Schroter and J. Schiller described the significance of chlorinated phospholipids as biomarkers of oxidative stress in various inflammatory diseases. Specifically, authors have nicely discussed the formation of chlorinated phospholipids, interaction with cellular biomolecules, and their involvement in various pathological conditions including atherosclerosis and arthritis. Most importantly, authors have discussed various mass spectrometry and chromatographic and immunological methods for the analysis of chlorinated phospholipids. Z. Qiao et al. in their review article discussed how dysfunctional autophagy is involved in the disease vitiligo by altering the redox homeostasis in the melanocytes. In this review, authors carefully discussed how oxidative stress and lipid oxidation could be involved in the pathophysiology of vitiligo. Further, they discussed how autophagy is regulated by the oxidative stress and its involvement in the melanocyte destruction leading to the vitiligo complication. The review articles in this special issue provide widespread information on the how oxidative stress and lipid aldehydes are involved in the human health and diseases.

The research article by M. Galicia-Moreno et al. investigates the role of oxidative stress markers in alcoholic liver cirrhosis patients. In this cross-sectional study comprising of 187 Latin-American patients, in addition to liver function tests, authors have also analyzed various oxidative stress markers in the blood such as lipid peroxidation, carbonylated protein and glutathione (GSH), and oxidized glutathione (GSSG). Interestingly they found that serum malondialdehyde, a marker for lipid peroxidation, levels are increased in proportion to the severity of the liver damage. At the same time, the levels of GSH and GSSG are varied according to the different stages of the liver cirrhosis. This study indicates that the increase in the oxidative stress markers was seen at the early stages of disease severity in alcoholic cirrhotic patients and abstinence from alcohol consumption restores GSH in patients with advanced disease severity.

Another cross-sectional study by S. Carrillo-Ibarra et al. reported the oxidative stress and inflammation status in patients with acute graft dysfunction (AGD) with Tacrolimus. In this study, authors have examined various oxidative stress markers such as lipid peroxidation and superoxide dismutase (SOD) and inflammatory markers such as TNF-alpha, IL-6, C-reactive protein, and nitric oxide in the serum. The results indicate that in patients with AGD there was an increase in the lipid peroxidation and levels of 8-isoprostanes and decrease in nitric oxide and SOD. However, no significance differences between serum TNF-alpha, IL-6, and C-reactive protein were observed in patients with or without AGD. These results suggest that deregulation of oxidative stress but not inflammation may be responsible for AGD.

A research study by H. Sonowal et al. examined the effect of aspalatone in preventing the endothelial dysfunction using cultured human aortic endothelial cells. Their results indicate that aspalatone prevents VEGF-induced lipid peroxidation in endothelial cells. Further, they have shown that aspalatone prevents VEGF-induced endothelial dysfunction as determined by examining the levels of eNOS, iNOS, ICAM-1, and VCAM-1. They have also shown that aspalatone prevents the VEGF-induced inflammatory response in endothelial cells. These results suggest a novel use of aspalatone in complications related to endothelial dysfunction.

In conclusion, we believe that our series of special issues on this research topic will continue to highlight the significance of lipid peroxidation products in human health and disease. A number of recent studies suggest that oxidative stress-generated lipid peroxidation products, which regulate cellular signaling pathways play a critical role in the pathophysiology of a number of disease complications. Several oxidative stress markers are now recognized as biomarkers of many human diseases and therapeutical approaches that potentially alter oxidative stress and generation of lipid peroxidation products are shown to be effective in controlling various human diseases.

